# Funding knowledgebases: Towards a sustainable funding model for the UniProt use case

**DOI:** 10.12688/f1000research.12989.2

**Published:** 2018-03-22

**Authors:** Chiara Gabella, Christine Durinx, Ron Appel

**Affiliations:** 1 ELIXIR-Switzerland, SIB Swiss Institute of Bioinformatics, Lausanne, 1015, Switzerland

**Keywords:** Bioinformatics, Data Resources, Knowledgebases, Funding, Long-Term Sustainability, Open Science

## Abstract

Millions of life scientists across the world rely on bioinformatics data resources for their research projects. Data resources can be very expensive, especially those with a high added value as the expert-curated knowledgebases. Despite the increasing need for such highly accurate and reliable sources of scientific information, most of them do not have secured funding over the near future and often depend on short-term grants that are much shorter than their planning horizon. Additionally, they are often evaluated as research projects rather than as research infrastructure components.

In this work, twelve funding models for data resources are described and applied on the case study of the Universal Protein Resource (UniProt), a key resource for protein sequences and functional information knowledge. We show that most of the models present inconsistencies with open access or equity policies, and that while some models do not allow to cover the total costs, they could potentially be used as a complementary income source.

We propose the *Infrastructure Model* as a sustainable and equitable model for all core data resources in the life sciences. With this model, funding agencies would set aside a fixed percentage of their research grant volumes, which would subsequently be redistributed to core data resources according to well-defined selection criteria. This model, compatible with the principles of open science, is in agreement with several international initiatives such as the Human Frontiers Science Program Organisation (HFSPO) and the OECD Global Science Forum (GSF) project. Here, we have estimated that less than 1% of the total amount dedicated to research grants in the life sciences would be sufficient to cover the costs of the core data resources worldwide, including both knowledgebases and deposition databases.

## 1 Introduction

### Knowledgebases, why?

Knowledgebases are organized and dynamic collections of information about a particular subject where data from multiple sources are not only archived, but also reviewed, distilled and manually annotated by experts. These digital infrastructures are essential to the effective functioning of scientific research and for the whole life science community: they serve as encyclopaedias, concentrating high quality knowledge collected from many different sources. In life sciences, knowledgebases are in general manually curated by experts, i.e. highly qualified scientists —called biocurators —who manually select, review and annotate the information on a particular subject. As a result, knowledgebases are collections of continuously updated data, providing a highly reliable source of scientific knowledge, with the data being validated and enhanced. There is a substantial difference between a repository and a knowledgebase. Both represent the computationally tractable accumulation of (pieces of) information and knowledge processed in such a way that the data is easily readable, understandable and exported. However, repositories rely partially or completely on data deposition by the users, while in knowledgebases, the information in general requires to be carefully selected and processed by experts. Both types of data resources are crucial for allowing research to be faster and more efficient as they:

- promote knowledge transfer to different sectors (e.g. between industry and academics),
- promote the re-use of the data, with new analysis/methodologies and comparisons,
- reduce the need to recreate or regenerate duplicate data,
- speed up research through easy access to integrated data, leading to considerable time and efficiency gains for researchers,
- make data available for teaching,
- generate scientific input and motivation for new research, by allowing scientists to apply computational methods to analyse new data in light of prior knowledge.

Despite the clear and increasing necessity for such high quality knowledgebases, the question of their sustainability in the long term is frequently raised, due to the current lack of an appropriate funding model. Sustainability is a major problem for all data resources: while many international initiatives are opened to discuss the sustainability of digital infrastructures, curated databases are often left aside (see Section 5 for a wider discussion on the existing initiatives and studies).

### Manual curation and open access

In life sciences, manual expert curation plays a fundamental role in the creation of high quality knowledgebases. Manual curation is acknowledged to be highly accurate ^1, 2^, but criticism is often raised about the necessity for such a time- (and cost-) consuming activity as opposed to the use of programs for automated or semi-automated information extraction (Information-Extraction programs—IE programs). In reality, current IE programs are not able to extract the large amount of information or compare data with the same accuracy as professional curators do, but they can be extremely useful for identifying mentions of single entities in the scientific publications, using for instance name-entity recognition tools ^1^. Consequently, manual curation cannot be fully replaced by the existing Artificial Intelligence (AI) technology. Text-mining is, however, often used as a first-line method for data extraction and identification of relevant literature.

The cost of professional curation is surprisingly low compared to the cost of open access journals’ publication charges, or to the cost of performing the related research. The Swiss National Science Foundation (SNSF) allows to claim CHF 3000 ^1^ (€2790) for costs of Open Access (OA) publication from agreed research funding. The Open Access Co-ordination Group in the UK estimates average fees at £1586 ^2^ (€1863). Per year, the curators of the UniProt knowledgebase, a key resource for protein sequences and functional information ^3^, read and/or evaluate between 50,000 and 70,000 papers, of which they fully curate approximately 8,000 publications. This means that in one year they read, evaluate and capture the output of research associated with OA publication costs of €100 to €200 million, significantly more than the budget of UniProt as a whole (∼ €15 million per year). Similarly, each publication that is read and/or evaluated, is the result of a research project grant with a typical value of ∼ $ 450,000 ^3^ (∼ €400,000). The cost of integrating the output (of the 8,000 publications) in UniProtKB, corresponds roughly to less than 0.1% of the cost to generate the research associated. In fact, a recent paper demonstrated that the costs of curation are quite modest on a per-article basis, and represent a fraction of the cost of the original research: the cost of biocuration of articles for the EcoCyc database is estimated at $ 219 (€193) per article over a 5-year period, corresponding to 6—15% of the cost of open-access publication fees for publishing biomedical articles, and to 0.088% of the cost of the overall research project associated ^4^. Additionally, a recent analysis on a curated knowledgebase showed that expert annotation is sustainable given that a large part of the literature is redundant and/or not relevant for the curation ^5^. Thus, curation costs are affordable in an absolute sense and represent a small fraction of the cost of the overall associated research projects that generated the experimental data.

Currently, most of the data resources are open access: their curated data are “digital online, free of charge, and free of most copyright and licensing restrictions”, i.e. without price barriers (subscriptions, licensing or pay-per-view fees) and permission barriers such as copyright and licensing restrictions ^6^. But open access is not to be confused with cost-free: making the data available involves significant labour, service and technology cost. Although there is likely scope for future costs containment of manual biocuration, a stable funding mechanism that ensures open data resources sustainability on the long term needs urgently to be established. In fact, while on the one hand the new techniques in machine learning and text mining are gradually improving the efficiency of automated information extraction programs, on the other expert curation will be always needed to guarantee the high quality of data through the selection and validation and the extraction of reliable information from published literature.

The European Commission policy on open access data is very clear:

*“The vision underlying the Commission’s strategy on open data and knowledge circulation is that information already paid for by the public purse should not be paid for again each time it is accessed or used, and that it should benefit European companies and citizens to the full. This means making publicly-funded scientific information available online, at no extra cost, to European researchers and citizens via sustainable e-infrastructures, also ensuring long-term access to avoid losing scientific information of unique value.”* ^4^

The scientific and political community is generally in favour of open access: data repositories/archives and knowledgebases mostly contain data produced through research work funded by public grants, and in principle, the information already paid for by the public purse should not be paid for again each time it is accessed or used. This is often the case for research articles, which are one of the primary media of knowledge dissemination. In many cases, papers in peer-reviewed journals are only available for a fee or, by open access charges. Moreover, data produced in research is not necessarily a finished product suitable for immediate usage or storage in data resources. So, dedicated funding is necessary to curate and structure the data so that they can be accessible and usable by the scientific community. This is not “paying again for already paid for information”. This is additional funding necessary to make the information accessible in a usable manner, so as to avoid additional, larger costs. Manually curated knowledgebases face the problem that they are insufficiently and unsustainably funded by public funds. The search for a sustainable funding model that ensures the maintenance and the future development of such resources remains thus a critical challenge. At the beginning of this millennium, a survey on existing databases reported that more than two-thirds (68%) of 153 considered databases had uncertain near futures (living expectation for 1–5 years of funding). Fifteen years later, only 24% of them were still alive (or rebranded) and 76% were no longer maintained, showing that a viable, sustainable framework for long-term data stewardship is sorely needed ^7^.

Until now, public knowledgebases have mainly been funded through institutional funding, user fees and/or research grants ^8^. The latter are grants intended specifically for research projects rather than infrastructures or databases. Funding through research grants is not an effective model on the long term, as it presents major limitations. Firstly, grants are competitive and they reward innovation: curated databases end up competing with innovative research projects (that, ironically, most often could not even be carried out without these databases). In addition, in order to obtain these grants that are focusing on innovation, databases and knowledgebases are typically pushed to adding new features, thus increasing the cost further. Secondly, grants are cyclic, with rounds of 3–5 years, with review criteria that are often not appropriate and applicable to infrastructures as they are conceived for research projects. Funds are thus not stable in the long term: often grants may not be renewed, or the funding for the renewed grant could be dramatically decreased. Alternatively, institutional funding could in principle guarantee the long term sustainability of the research infrastructures as contracts are often negotiated and fixed over several years, allowing data centres to plan in advance and build the infrastructures. At the same time, the weakness of such model lies indeed in its inflexibility, which may not always allow to keep the pace with the growing data volume and improving techniques. Also, data access charges through subscription or user fees remain incompatible with the rising principles of open access. For these reasons, it is very common to see data resources combining the longer-term, but rather inflexible, institutional funding, with more flexible shorter-term research grants. It is important to mention that many data resources depend on research grants particularly in their early stages, as they are often the result of a research study. Yet, while this funding model often allows to identify the need for certain resources in the scientific communities, it is not intended to sustain them on the long term, inconsistently with the long living scope of such resources.

## 2 Overview of existing funding models

In this paper, twelve funding model have been identified and are described here below. They include existing funding models for data resources and facilities, as well as possible scenarios that are currently considered by various international initiatives. For each model, some examples of existing data resources funded through that mechanism are listed. It has to be noticed that all the examples presented in this paper do not depend exclusively on one funding mechanism. In general, a data resource combines several revenue streams, in order to differentiate the income sources. The list here below is though not exhaustive, but introduces the major funding sources in the various sectors of research, with a particular focus on the life sciences. Some funding models that are currently not specifically supporting data resources are also included, as they could be implemented for data stewardship and preservation.

**Figure 1.**
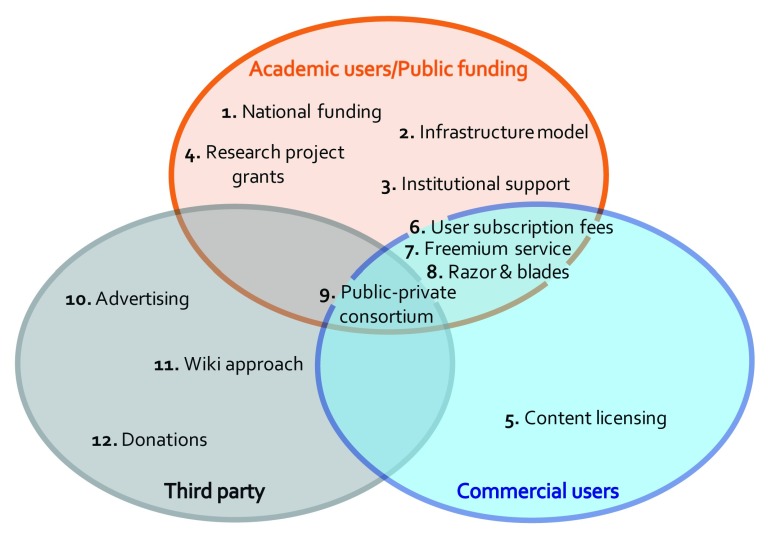
Funding models sources. The 12 considered models are represented depending on the origin of the revenues.

The twelve models can be grouped in three main categories depending on the revenue origins ( Figure 1). Most of the models rely on funds coming from national budgets and allocated to research and/or infrastructure, redistributed among the applicants, institutions or services according to various rules and conditions. A second category embraces all the models dependent on user fees. Finally, there are models counting on voluntary donations and participations, or third party funding. On top of these, models exist that are a mixture of these categories, as they benefit together from national funding and commercial fees or investments, or they take advantage of funding from government bodies, industries and voluntary donation.

1. **National funding:** governmental agencies fund the infrastructure directly, through non-cyclical funding programmes. For research infrastructures, funds derive directly from the domestic R&D budgets. Often, users are charged for some subscriptions or special services. Examples are:

- National archives, libraries such as the National Library of Medicine ( www.nlm.nih.gov) at the National Institutes of Health (NIH), statistical agencies;
- NASA archives, State archives;
- Public universities.

2. **Infrastructure model** ^9^: funding agencies pay directly for data resources as a necessary part of the research infrastructure, through a percentage of the research funding that is specifically set aside. The grants themselves are only allocated to research projects. A percentage of each grant is then retained and assigned to a budget for data stewardship, and subsequently redistributed among the relevant infrastructures, including knowledgebases. This model is similar to the *National model* (model 1), but in this case funding agencies are not necessarily national (they can also be private, thus with different budget constraints). The funding agencies contribute financially in proportion to the grant volume that they allocate to research. This model is not implemented yet as a funding model for life sciences knowledgebases.

3. **Institutional support:** universities or institutions have their own repository/data bank that is maintained through the “internal” institutional funds. Grants can be cyclic or long-term, and usage may be restricted to the institution’s members or be open to the worldwide community.

- It is often used to support specialist resources, such as CAZY ( www.cazy.org) —the Carbohydrate-Active enZYmes Database, funded through the French National Center for Scientific Research (CNRS) and the Aix-Marseille University
- UniProt ( www.uniprot.org) is partly institutionally funded through the SIB Swiss Institute of Bioinformatics (with governmental funding) and the European Bioinformatics Institute (EMBL-EBI) (with member states funding).

4. **Research project grants:** competitive cyclic research or dedicated resource grants from national funding agencies such as the NIH, the National Science Foundation (NSF) or the Swiss National Science Foundation (SNSF). They request a submission by the applicant every 3–5 years. Access is free for the user. This category includes also a few existing grants specifically conceived for databases and resources ^5^. Most of the databases and knowledgebases in the life sciences are supported by these type of grants, including:

- FlyBase ( flybase.org) —database for Drosophila genetics and molecular biology: grants from the National Human Genome Research Institute (NHGRI) at the NIH. Support is also provided by the British Medical Research Council, the Indiana Genomics Initiative, and the NSF;
- ZFIN ( zfin.org) —Zebrafish Model Organism Database: NHGRI and small amounts from NSF;
- MGI ( www.informatics.jax.org) —Mouse Genome Informatics : NIH grants;
- RGD ( rgd.mcw.edu) —Rat Genome Database: NIH grant;
- TAIR ( www.arabidopsis.org) —The Arabidopsis Information Resource, from 1999 to 2013: NSF grant;
- PeptideAtlas ( www.peptideatlas.org) —database of re-analysed Mass Spectrometry peptides identification: grants from the European Commission and three institutes of the NIH;
- RCSB Protein Data Bank ( www.rcsb.org) —the macromolecular 3D structure database: grants from seven federal sponsors through the wwPDB organization of four international partners.

5. **Content licensing/industrial support model** ^10^: requires commercial users to pay a fee for access to the data and for-profit reuse, whereas data are free for non-commercial users.

- Between 1998 and 2004, for-profit users were paying an annual fee for access to Swiss-Prot (now part of UniProt), whereas academic researchers had free access. Swiss-Prot returned to an all-user-free access model in 2004 after the SIB Swiss Institute of Bioinformatics, the European Bioinformatics Institute (EMBL-EBI), and the Protein Information Resource (PIR) formed the UniProt consortium and obtained a grant from the NIH. For more details on this case study, see Section 3.

6. **User subscription fees:** users are charged on a time base (e.g. every month or year) or on download sizes, and they have access to the entire database. At the end of the validityA, the subscription must be renewed to continue the access.

- Many scientific journals, including prestigious ones, such as Nature, Science or Cell;
- KEGG ( www.genome.jp/kegg), the Kyoto Encyclopedia of Genes and Genomes: a pathway database;
- TAIR, since 2013: curators formed a non-profit company (Phoenix Bioinformatics) and since then it mostly relies on tiered subscription revenues (national, institutional or individual subscriptions) ^11^. So far this model has been described as successful in maintaining the database’s quality and user base ^12^.

7. **Value-added/asymmetrical pricing model (freemium service)** ^8^: a basic data set within the database is freely available to anyone. Individual scientists or companies that are willing and able to pay a higher fee can buy additional levels of service, better data access or additional tools and resources.

- TRANSFAC ( gene-regulation.com/pub/databases.html) ^13^ —knowledgebase of eukaryotic transcription factors and their regulated genes: has a free public version dated 2005, while the professional version, that is susceptible to subscription to provide full access, is regularly updated and presents more advanced tools and an easy-to-use interface;
- The Cambridge Crystallography Data Centre (CCDC) with the Cambridge Structural Database (CSD - www.ccdc.cam.ac.uk) —the small molecule crystallography data knowledgebase;

8. **Infrastructural razor & blades** ^14^: an attractive, inexpensive or free initial offer (“razor”) encourages continuing future purchases of follow-up items or services (“blades”).

- Applied to the public sector information environment, this model sees datasets stored for free on cloud computing platforms and accessible by everyone via APIs (“razor”). Re-users are charged only for the computing power that they employ on-demand (“blades”). Application of this model is limited to contexts and domains in which the computational costs to access the datasets are significant;
- GENEINVESTIGATOR ( genevestigator.com) —search engine for gene expression: 7-days free access to the professional edition and permanent free access to the Basic edition for academics.

9. **Public-private consortium** ^7^: is a mixture of funding from government bodies and industries. The funders mandate the research subjects and supporting companies do not receive priority access to data.

- The SGC ( www.thesgc.org) —Structural Genomic Consortium: it consists of three academic laboratories in Oxford, Toronto and Stockholm and is funded by a consortium of 13 public and private bodies including GlaxoSmithKline, Genome Canada, Merck, Novartis, the Swedish Foundation for Strategic Research and the Wellcome Trust. The three laboratories solve protein structures chosen by the funders. All solved structures are deposited in a data bank, but supporting companies do not benefit of priority access.

10. **Online advertising and corporate sponsorship:** corporate sponsorship is part advertising and part dealmaking —the corporation pays to support a database that provides value to its potential customers.

- GeneCards ( www.genecards.org) —database of human genes that provides genomic, proteomic, transcriptomic, genetic and functional information on all known and predicted human genes: it is free for academic non-profit institutions; other users need a commercial license. Advertisings appear on the website as banner ads. However, the income from advertising does not allow GeneCards to be self-sustainable: other funds come from academic grants and database licences’ royalties.

11. **Open source volunteering** (or **wiki approach**) ^15^: replacing part of data curation by community participation can be attractive as it has a low cost. It depends, however, on drawing contributions from busy users. In addition, contributions tend to be sporadic, leaving many gaps. Hence it can only replace (a small) part of curation and therefore still requires funding for curation, software engineers, storage space, and operating costs.

- GeneWiki ( en.wikipedia.org/wiki/Portal:Gene_Wiki) —informal collection of pages on human genes and proteins;
- WikiProteins —web-based, interactive and semantically supported workspace based on Wiki pages of biomedical concepts ^16^;
- TOPSAN ( proteins.burnham.org) —a collaborative annotation environment for structural genomics ^17^.

12. **Donations**: philanthropic funding such as grants and donations can generate income. They partly depend on the impact on and awareness of the (user) population.

- Human Protein Atlas ( www.proteinatlas.org) —funded by the Knut & Alice Wallenberg Foundation;
- Human Cell Atlas ( www.humancellatlas.org) —funded by the Chan Zuckerberg Initiative;
- Wikipedia ( www.wikipedia.org) —funded by small voluntary donations from thousands of users.

13. **Mixed models:** as mentioned, most of the knowledgebases rely on **diversified** multiple funding streams. This approach has the obvious advantage of increasing resilience if one of the sources disappears. Some example of databases or knowledgebases supported by a mixed model are:

- UniProtKB: Swiss government through the SIB Swiss Institute of Bioinformatics (4-year grant), NIH (4-year grant) and EMBL-EBI;
- PRIDE ( www.ebi.ac.uk/pride) —PRoteomics IDEntification database, part of ProteomeXchange: 25% EMBL-EBI, 50% Wellcome Trust (5-year grant), and 25% UK Biotechnology and Biological Sciences Research Council (BBSRC - research infrastructure grant);
- OMIM ( www.omim.org) —Online Mendelian Inheritance in Man, a catalogue of human genes and genetic disorders, with a particular focus on the gene-phenotype relationship: NIH and, also, very recently through donations;
- InterPro ( www.ebi.ac.uk/interpro) —database for protein sequence analysis and classification: EMBL-EBI, BBSRC and Wellcome Trust;
- Ensembl ( www.ensembl.org) —genome database and browser for the retrieval of genomic information: Wellcome Trust, NIH, EU FP7 and EMBL-EBI;
- Europe PMC ( europepmc.org) —on-line database of free access biomedical and life sciences research literature: managed and developed by the EMBL-EBI on behalf of an alliance of 26 research funders, led by the Wellcome Trust;
- TAIR: user subscription fees (national, academic institutional, individual, and corporate subscribers) and a grant from the SLOAN Foundation. There are also elements of the *Freemium model* as non-subscribers have some free page views before encountering a monthly limit.

The models are summarized for comparison in Table 1. Each model is described in terms of its compatibility with open access policies and its equity among the potential users and institutions, i.e. whether or not wealthier institutions or certain users are particularly favoured. Also, the forecasted stability of the models over time and the key dependency of each funding stream are indicated. Associated factors such as national/international economic situation dependency (which are obviously relevant within each model described) has been indicated only when representing the main dependency. The dependency of the funding is crucial to describe the vulnerability of the models and also needs to be taken into account when setting up a mixed model. The best funding model would combine models that are dependent on different factors.

**Table 1.** Comparison of the 12 models in function of open access, equity, stability and key dependency. The aspects that favour open access, equity of users and stability over time are highlighted in bold.

#	Name of the model	Compatible with open access?	Potential for equity of users or institutions	Stability forecasted over time	Key dependency
1	**National funding**	**Yes**	**High**	**Stable**	National economic situation
2	**Infrastructure model**	**Yes**	**High**	**Stable**	Research spending by funding agencies
3	**Institutional support**	**Yes**	**High**	**Stable** or Cyclic	Institutional funds availability
4	**Research project grants**	**Yes**	**High**	Cyclic - grants renew every 3–5 years	Infrastructure/research spending by funding agencies
5	**Content licensing/industrial** **support model**	No	Low	Function of usage	Commercial partner
6	**User subscription fees**	No	Low	Function of usage	Usage
7	**Value-added/asymmetrical** **pricing model (or freemium** **service)**	Not completely	Low	Function of usage	Usage
8	**Infrastructural razor & blades**	No	Low	Function of usage	Usage
9	**Public-private consortium**	**Yes**	**High**	**Potentially stable**	Commercial partner
10	**Online advertising &** **Corporate sponsorship**	**Yes**	**High**	Function of usage	Usage, commercial partners
11	**Open source volunteer (wiki** **approach)**	**Yes**	**High**	Highly dependent on participation	Willingness to contribute
12	**Donations**	**Yes**	**High**	**Potentially stable**	Partners

## 3 Funding situation of the UniProt knowledgebase, past and present

For the purpose of this work, the Universal Protein Resource (UniProt) knowledgebase is used as a case study. UniProt contains a reviewed collection of high-quality annotated and non-redundant protein sequences, and brings together experimental results, computed features and scientific conclusions. Expert curation constitutes a core activity in the development and maintenance of the UniProt Knowledgebase (UniProtKB), which is composed of UniProtKB/Swiss-Prot - the reviewed section containing expert curated records with information extracted from the literature and curator-evaluated computational analysis, and UniProtKB/TrEMBL - the unreviewed section with automatically annotated records. At present, UniProt is developed and maintained by the UniProt consortium, a collaboration between the SIB Swiss Institute of Bioinformatics, the European Bioinformatics Institute (EMBL-EBI), and the Protein Information Resource (PIR). UniProt also includes the UniProt Reference Clusters (UniRef), a database of clustered sets of sequences from the UniProtKB, and the UniProt Archive (UniParc) that provides a complete set of known sequences, including historical obsolete sequences.

The UniProt knowledgebase is an interesting case study because it passed through various funding models, as well described in the literature ^7, 18, 19^. It started under the name of Swiss-Prot, a research project at the University of Geneva in 1986. At that time it was funded through a Swiss National Science Foundation (SNSF) research grant, which lasted until 1996, when the knowledgebase suffered a funding crisis ^20, 21^. After negotiations with the Swiss Government, an agreement was reached with the creation of an institutional framework for the knowledgebase: the SIB Swiss Institute of Bioinformatics, born on 30 March 1998 as a non-profit foundation that could fund 50% of the knowledgebase. Simultaneously to SIB, the company Geneva Bioinformatics (GeneBio) S.A. was established as the exclusive commercial representative of SIB, to compensate the other 50% of the costs. GeneBio was selling licenses to commercial users, the fee depending on the number of users in the company, while academics users had free access. The royalties greatly exceeded the portion of the budget provided by the Swiss Federal Government. The Swiss-Prot group grew rapidly to the size of 80 people in 2004, while the database more than quadrupled in content in 6 years. In 2002, the funding model of Swiss-Prot changed and returned freely accessible to all the users. SIB and EMBL-EBI joined with PIR to form the UniProt consortium and applied for a NIH grant. Today the UniProt consortium has three main funders: the Swiss government (from 1996 and through SIB from 1998), recently with a 4-year grant from 2017 to 2020 accounting for about 38.7% of the total costs, an NIH grant ending in April 2018 (∼ 32.5%), funds from the European Molecular Biology Laboratory (EMBL, ∼ 25.4%) and other funding of different sources (∼ 3.4%). Swiss-Prot has also been supported by some EU funding, which ended in 2009. Now, despite the fact that about 28% of its users are from Europe, only a little portion of the curated part of the UniProtKB is currently supported by European funding: the UniProtKB/Swiss-Prot efforts in Switzerland are exclusively funded by Swiss and US funds, while the resource is being used by researchers all over the world.

The yearly total income of UniProt is in the order of $ 17 million (∼ €15 million), of which more than 90% is going to the staff salaries. This income hardly allows the curation of the current relevant literature ^5^. However, it does not allow any expansion that is required by the fast-growing need of literature biocuration

## 4 Application of the models to the UniProt case

In this section, the models presented in Section 2 are applied to the case study of the UniProt knowledgebase. For each model, the conditions to obtain an income equivalent to the UniProt annual effective costs, rounded up to €20 million, are analysed.

When possible, the analysis of the feasibility of these models is extended to a theoretical global cost of the ensemble of the bioinformatics major core data resources for life science research (i.e. repositories and knowledgebases), estimated to about €190 million. By extension and to simplify the reading, this amount will be referred to as the budget for the “total core data resources”. This value has been assessed from an estimated cost of the ELIXIR candidate core data resources (∼ €70 million ^6^, per 435 million inhabitants for the ELIXIR’s Member States), extrapolated to a virtual geographical area that includes Europe, USA and Japan (respectively of 743 million, 320 million and 128 million inhabitants, for a total of 1.19 billion inhabitants). Other studies ^22, 23^ reported different values for other groups of resources and / or infrastructure, which may be in contrast with the data presented here. Yet, it is important to emphasize that the amounts presented in this work are rough estimations and should not be taken as financial reference data: their main purpose is to illustrate how the funding models could be applied to a real case study.

All estimations are based on available data (number of users, data download, ads, etc.). The revenue values are not intended to be an exact calculation, but should be used as an indicator of the income potential for the different models. Usage data of UniProt have been obtained through Google Analytics, with adjustments for the user population as in 23, 24. This triangulation leads to an estimation of unique users per month at 83,000 units.

Whenever possible, data are presented in the original currency from which they are derived, transformed in euros for ease of understanding (US$ 1 = €0.88, CHF 1 = €0.93, currency rates as at July 2017). To simplify the reading, amounts are approximated and each model is considered as a single model (i.e. no mixed model). The depth of the analysis of each model depends in general on its applicability to the UniProt case study. Some models that are theoretically applicable, but dependent on several variable parameters, are also not presented in greater detail as they would require a separate business analysis that is out of the scope of the present study. Also note that the analyses are performed under the hypothesis that the choice of the model does not influence the parameters of the same model (i.e. no feedback loop).

**1. National funding.** In this model, the countries having the highest access rates of the UniProt website would pay an amount to the UniProt consortium, proportional to the usage (2016 data) or to the national wealth (OECD data ^7^). Four parameters have been separately taken into account for this analysis: the UniProt usage rate, the Gross Domestic Product (GDP), the Net National Income (NNI) and the R&D domestic spending. Table 2 gives an overview of the costs for the top-10 user countries, both for the UniProt case and for the total core data resources. For the first parameter, the costs respectively for UniProt and for the total core data resources are distributed among the user countries according to their usage rate. A very small percentage of the national budget (0.00025–0.00035%o) or the R&D spending (0.014%o) would allow the sustainability of UniProt. Similarly, supposing the same geographical usage distribution for total core data resources infrastructure as for UniProt, it is possible to estimate that 0.0024–0.003%o of the total budget or 0.13%o of the total R&D spending could sustain the total core data resources.

**Table 2.** Model 1, National funding. Potential amounts from the top-10 UniProt user countries to sustain UniProt (orange columns) and the total core data resources (blue columns). Costs per country as a function of (1) usage, (2) Gross Domestic Product (GDP), (3) Net National Income (NNI) and (4) R&D domestic spending.

	Country	% of usage	UniProt				Total core data resources			
			Tax based on usage [k€]	0.00025 ‰ of GDP [k€]	0.00035 ‰ of NNI [k€]	0.014 ‰ of R&D spending [k€]	Tax based on usage [k€]	0.0024 ‰ of GDP [k€]	0.003 ‰ of NNI [k€]	0.13 ‰ of R&D spending [k€]
1	United States	26.64	5,862	4,163	4,538	5,693	53,288	39,965	42,548	52,862
2	China	9.72	2,138	4,476	2,634	4,349	19,438	42,966	24,697	40,384
3	United Kingdom	6.87	1,512	639	675	533	13,741	6,139	6,332	4,950
4	Germany	6.10	1,342	923	964	1,285	12,201	8,857	9,036	11,929
5	India	5.47	1,204	1,838	2,353	875	10,944	17,648	22,060	8,126
6	Japan	4.35	958	1,130	1,187	2,064	8,706	10,852	11,131	19,170
7	France	3.26	717	641	669	712	6,515	6,153	6,270	6,610
8	Canada	2.69	592	374	389	321	5,385	3,595	3,644	2,985
9	Spain	2.27	500	373	386	236	4,546	3,583	3,617	2,192
10	Italy	1.96	431	524	543	337	3,916	5,034	5,089	3,130
…	…									
14	Switzerland	1.49	328	120	122	162	2,986	1,149	1,142	1,500
…	…									
	Total	100	€20 million				€190 million			

This model guarantees secure funds for knowledgebases, and is stable over time. It is compatible with the criteria of open access and equity for users and institutions, and coherent with the idea that the countries with the highest number of users contribute to the maintenance of the infrastructures from which they are benefitting. A contribution based on the R&D spending might be preferred to the GDP as these two do not always correlate. For a country with a large population such as India, where the expenditures in research represent only the 0.85% of the GDP (compared to 3.2% for Japan, for example), the contribution might be seen as unfairly large for the government.

At the international level, some recommendations that are consistent with this model have been recently put forward. The European Commission’s High Level Expert Group on the European Open Science Cloud (HLEG - EOSC) proposed that about 5% of the total research expenditure should be spent on properly managing and ‘stewarding’ data in an integrated fashion. The implementation of this model requires, however, that the different governments or national funding agencies agree to contribute with a fixed percentage of their R&D budgets, which in general cover other research domains other than the life sciences. This model can therefore not be put into place in a short timeframe, and governance costs may represent a considerable fraction of the funds obtained.

**2. Infrastructure model.** The cost of data stewardship would be covered directly by the funding agencies that fund field-related research projects. This model can be implemented as a sort of revised version of *National model*, model 1: funding bodies (not only governmental, but also private agencies) allocate a fixed percentage of their life science grants to a budget that is subsequently distributed to the infrastructures, knowledgebases included, according to well-defined selection criteria. To estimate the percentage needed to sustain UniProt and, more generally the total core data resources, the budgets reserved to the life sciences from five theoretically selected funding agencies, have been considered (the Swiss National Science Foundation ^8^, the National Institutes of Health (NIH) ^9^, the Wellcome Trust ^10^, the Japan Science and Technology Agency ^11^, and the European Commission ^12^. The total yearly budget assigned to the life sciences by these five agencies adds up to ∼ €21 billion. A very small fraction of this budget, in the order of 0.1%, would then be sufficient to sustain UniProt. **Only 1%** of the total amount dedicated by these five funding bodies to grants in the life sciences would suffice to cover the cost of the total core data resources (0.9% of these budgets corresponds to approximately €190 million). Extending this model to other major funding agencies would increase the income and reduce the percentage needed from each agency.

This approach is very attractive in terms of equity and potential of income. It requires the major funding agencies to collaborate at an international level, and represents a significant evolution in the way how research infrastructure is funded. It also necessitates that countries that are currently not funding life science databases, or in a small proportion compared to their usage, start contributing. In general, funding agencies’ revenues can come from different sources, not only from the national budget (as in model 1); therefore, the participation of a certain funding agency to this model would likely depend on the availability of its own local budget, while the identification of the knowledgebases to which the budgets are allocated would require a selection process based on well-defined indicators (as it is currently done for grant assignments) and a lead agency or an institution that would take care of the funding distribution process. See Section 5 for an in-depth discussion about this model.

**3/4. Institutional support + research project grants.** These two models are equivalent to the current funding scheme of UniProt, with 63% of the budget covered by institutional support (from Switzerland through SIB and from the EU through the EMBL-EBI), and 32% by NIH funding, granted for four years until 30 April 2018.

**5. Content licensing.** UniProt is used by many life science companies to carry out business, research and to generate profit. The potential income of a commercial paywall is thus estimated, by assuming that all the life science for-profit companies would subscribe a licence to UniProt. A (non-exhaustive) list of the life science companies of 30 major countries in the world, irrespective of their size, was extracted ^13^ together with a classification of all manufacturing companies, in terms of their size ^14^. By assuming that the relative proportions of small, medium and large companies in the life sciences sector are similar to the proportions in the whole manufacturing area, the distribution of the life science companies in terms of their size was estimated. In this case, even low licence prices would generate an income of about €20 million, e.g.:

- €500 to small companies (0–9 employees)
- €1,000 to companies of 10–19 employees
- €3,000 to companies of 20–49 employees
- €5,000 to companies of 50–249 employees
- €10,000 to companies of 250+ employees.

The licence prices were intentionally underestimated in order to balance with the overestimation of the number of subscribing companies (>15,000).

When this model was applied to Swiss-Prot in 1998, the licence fees ranged from €2,500 for small companies (typically start-ups) to €90,000 for the largest companies. At that time, the necessary annual budget for Swiss-Prot was ∼ €8 million. When calculating the revenues using these licence fees, a subscription by 1/10 of the total companies calculated above, would allow for a sustainable model for the knowledgebase. The implementation of this model requires some extra administrative costs, such as the creation of an adequate platform for the payment of the licences (probably on the order of few FTEs) and the costs for negotiation with the companies. This model has a large potential of income and allows maintaining a free access to the knowledgebase for academic users, while not for commercial users. It is though not compatible with the principles of open access and could hamper licensing and reuse of data by other resources that might see their access limited or blocked.

**6. User subscription fees.** As estimated, UniProt has a traffic of about 83,000 unique users/month and average monthly data download from the FTP site of 30 TB. Charging the single user with a subscription fee of €20/month would allow an income sufficient to sustain the resource. Similarly, charging the user according to data download a fee of €0,055/MB download, would cover the yearly budget of €20 million. While these amounts are comparable to many subscriptions for software or applications, this model remains inconsistent in terms of equity and open science. Moreover, the implementation of this model would also require the setup of a platform for the payments, or the adoption of an existing one, with some additional (but probably negligible) costs. As the previous model, it is not compatible with the principles of open access.

**7/8. Freemium service / Razor & blades.** The potential income for UniProt through these two models is difficult to estimate. Their implementation would imply that a selected part of the information (or old releases) was available for free and additional features (or the latest releases) were dependent on the payment of a fee. The data within UniProt would therefore have to be split into “free" and “not-free-but-worth-paying-for", in terms of data selection or old/new releases, which would require a more in-depth analysis and a careful selection of the type of data to charge and release. This model is currently used by some scientific journals such as the Proceedings of the National Academy of Sciences (PNAS): access to the complete PNAS Online is limited to paying subscribers and to members; without a subscription, all content older than 6 months is accessible at no cost. Similarly, TAIR adopted the same policy: up-to-date curated data are available to subscribers and one year later they become freely available for anyone to download. Interestingly, from an early analysis, the group reported that the introduction of a paywall did not decrease the use of the database ^11^. These models, however, are not compatible with the principles of open access and they also require an infrastructure comparable to the subscription model to support the fee services. Moreover, they cannot guarantee the long term survival if all the resources will have to rely on subscription fees: a paywall for accessing each resource will heavily charge the user that will inevitably choose to dismiss some of them.

**9. Public-private consortium.** A biotechnology/pharma company consortium financing UniProt is an option that would allow academic users a free access, with the budget of the resource being supported by a consortium of companies that make use of the knowledgebase. Yet, it is hard to estimate how many and which companies would be willing (or able) to participate, among which the cost (or part of it) would be distributed. A similar model to cover part of the costs could also in principle see field-specific companies funding the part of UniProt aligned with their interests, but without a privileged access to the data. In this way, the resource would remain open access for the users, although funded by private companies. However, the history of Swiss-Prot has shown that commercial users prefer to pay a (compulsory) licence subscription because a voluntary contribution is not easily defendable in the annual budget. The recent experience of the TAIR knowledgebase also shows that support from companies as a voluntary participation lags behind mandatory fees ^11^.

**10. Advertising.** This model could in principle be applied to UniProt in many different manners. One possibility is to have banner ads of related companies on the web page sides proposing pharmaceutical products, lab tools, antibodies, reagents, etc. This option has the inconvenience that the advertisements may damage the high quality image of the database, in addition to being intrusive and annoying for the users. A second possibility may be to add links to company websites that are selling products related to the proteins findable through the UniProt search tool. Also, the addition of some sponsor services, as for instance the inclusion of a comparative table of products, can be of added value to the knowledgebase. Another possibility is to collect users’ data (e-mails, searches, locations . . .) and to exchange - provided permission from the users is obtained - the information with advertisers or partners. This is a model adopted by services as Google, Facebook and Apple, and by scientific journals such as Nature and Science. This model raises criticisms in terms of privacy and high scientific quality of the database. Moreover, the setup of an advertising platform is associated with additional costs and staff to support the structure. An advertising model requires a high volume of visitors to provide a sustainable income. To generate $ 50,000 (€44,000) per year in advertising revenues, a website needs approximately 2 million page visits per year ^10^ The UniProt traffic of about 56 million page views per year could generate a maximum of €1.3 million, less than 1/15 of the annual budget. This model can therefore not be the unique funding source for UniProt, but rather a complementary stream to other models, as compatible with the principle of open access.

**11. Wiki approach.** The model does not generate a revenue, but describes a way how data could be annotated, i.e. through voluntary participation of the community. The added value of UniProt lies in the high quality of the annotated data, curated by professional expert biocurators. Public contributions cannot maintain this high level of accuracy of the data. There exist many examples of resources that adopted a “Wiki-based” approach, in genomics (GeneWiki, WikiGenes), in proteomics (WikiProteins, TOPSAN), as well as for RNA annotation (Rfam, miRBase). However, these systems still encounter many obstacles such as usability, authorship recognition, and information reliability ^25^. In addition, many of these Wiki resources are based on the integration of already processed data collected from existing knowledgebases such as UniProt. New approaches have been presented to increase reliability and usability, such as mechanisms to track authorship and to encourage community participation ^25^, but many issues remain to be addressed. For example, a Wiki approach still requires funds for the basic infrastructure (servers, technical staff, . . . ). It could therefore be implemented, combined with other more robust models, as a solution to reduce the total cost of the knowledgebase. Studies have evaluated the multiple attempts to take advantage of the significant experience of the life science community (passionate scientists, students, retired researchers, . . .) through some sort of crowd-sourcing. Yet, crowd-sourced curation appears to have a very low participation rate. In general, the more complex a database is, the more likely professional curation is to be favoured over crowd-sourced curation ^22, 26^. Therefore, this approach is definitely not applicable to the case of UniProt: high quality data thanks to professional expert curation are at the heart of this resource, and quality could not be guaranteed through a crowd-sourced curation.

**12. Donations.** Many online databases and journals rely on donations from people around the world. The most famous is Wikipedia, the free collaborative collection of knowledge. Even though Wikipedia’s content comes from active users on a voluntary base (i.e. at cost zero), the site has running costs that are covered primarily by individual donations, in addition to other funding sources that allow to sustain specific projects. In 2016, the Wikimedia Foundation received $ 77.2 million (€72.5 million) from 5.4 million users (∼ 1% per year of all users, with an average donation of about $ 15 ∼ €13) ^15^. By applying a similar model to the UniProt case, the same fraction of users could potentially contribute with similar donations to the knowledgebase. However, donations would contribute to less than 1% of Uniprot’s budget (∼ €115,000). Also, this model is highly unpredictable, as donations depend on individuals, the awareness of the funders and a strong involvement in the cause. Moreover, some extra costs are to be included, as such a model requires setting up a fundraising infrastructure (people, campaign, department, . . .). Yet, it is worth considering this model as a complementary model.

## 5 Discussion and proposal for a long-term sustainable funding model for knowledgebases

As described above, most life science knowledgebases are currently heavily dependent on grants and paid subscriptions: these funding models present many limitations that are described in Section 2. The ideal funding model for UniProt, with possible extensions to the total core data resources, should respond to the following criteria:

- To guarantee open access and equal opportunity
- To generate revenues that are sufficient to fully cover the costs and that are stable over time
- To derive from transparent sources
- To combine different revenue streams, in order to reduce the risk of lacking income if one of the sources is discontinued; the different revenue streams have to depend on different external factors or different entities (see Table 1) to further increase resilience.

Table 3 summarizes the pros and cons for each model, with a focus on UniProt, and an estimation of the time frame necessary for its implementation. A complex model would obviously require a longer period of time to be put in place and accepted by the community.

**Table 3.** Applicability of the models to the UniProt case study. The table summarizes the potential of income of each model and the complexity of the implementation. Refer to Section 5 for the calculations.

#	Name of the model	Applicable to UniProt?	Potential and condition for income for UniProt	Estimated implementation time	Pros (+)	Cons (-)
1	**National funding**	Yes	0.00025–0.00035‰ of the domestic budget from each of the user countries, or 0.013‰ of the R&D domestic spending, allow covering 100% of the budget (€20 million)	Several years	+ Stable funding in the long term + Open Access	- Requires negotiation with the respective governments
2	**Infrastructure model**	Yes	∼ 0.1% of total spending for life science research grants of 5 funding agencies allow covering 100% of the budget	Months to years	+ Stable funding in the long term + Open Access	- Requires negotiation with the funding agencies (but likely a smaller effort than in *model 1*)
3	**Institutional support**	Current (SIB & EMBL-EBI funds)	63% of the budget	Already existing	+ Funding relatively stable over time seen the institutional commitment + Open Access	- Amounts insufficient to cover full cost
4	**Research project** **grants**	Current (NIH grant)	32% of the budget	Already existing	+ Open Access	- Amount insufficient to cover full cost - Short funding cycle leading to instability over time
5	**Content licensing/** **industrial support** **model**	Yes	Commercial licences for private companies (€500 – €10,000: depending on the size) allow covering 100% of the budget	Months	+ Stable funding in the long term + Potential high income	- Not Open Access - Significant administrative burden
6	**User subscription** **fees**	Yes	Subscription fees for users of €20/month or €55/GB of download allow covering 100% of the budget	Months	- Stable funding in the long term + Potential for high income	- Not Open Access - Significant administrative burden
7	**Value-added/** **asymmetrical pricing** **model (or freemium** **service)**	?	?	Months	+ Potential for high income	- Has to be combined with another model - Not Open Access - Significant administrative burden
8	**Infrastructural razor** **& blades**	?	?	Months	Potential for high income	- Has to be combined with another model - Not Open Access - Significant administrative burden
9	**Public-private** **consortium)**	Yes	Consortium sharing the costs size allows covering up to 100% of the budget	Months to years	+ Potential for high income + Stable funding in the long term + Open Access	- Requires negotiation with the companies
10	**Online advertising** **& Corporate** **sponsorship**	Yes	€1.3 million (6,5% of the budget)	Months	+ Open Access	- Has to be combined with another model - May decrease the scientific credibility & be annoying to the user - Significant administrative and business development effort
11	**Open source** **volunteer (wiki** **approach)**	No	-	Months	+ Open Access + Can be used to decrease the total costs	- Has to be combined with another model - May decrease the scientific credibility and quality
12	**Donations**	Yes	Voluntary donation of *sim* €13 from 1% of the users could cover <1% of the budget	Months	+ Open Access	- Highly unpredictable - Has to be combined with another model - Requires an adequate platform for donations and significant fundraising efforts

A model relying on access fees (model 5, *User subscription fees*, as well as model 6, *Content licensing*) would likely guarantee the sustainability of UniProt, at least as long as the resource remains useful and has an impact for the community. If academics could have a privileged free-of-charge access, commercial entities would contribute financially to the maintenance of the knowledgebase through a subscription fee. The introduction of a paywall, even for a part of the users, would probably impact data reuse, access and submission. One of the principal concerns is that a paywall may prevent researchers from linking to data in other databases. And of course, scientists would need to use their grant money to pay for subscriptions. One option to recoup the usage costs could be a “virtual coins model”, in which the costs for using the resources is included directly in the grant applications and a virtual budget is assigned specifically for that purpose. In this way, the resource is maintained as long as it is sufficiently used and the researchers receive pre-paid credits to access the infrastructure. In theory, the difference with a subscription fees model is that the user doesn’t subtract part of his research budget to pay the infrastructure, as the amount that s/he needs to pay is foreseen in the project estimates. The closest scenario is perhaps the BD2K Cloud Model ^16^, though it relates to data storage and deposition databases. It hardly applies to resources such as knowledgebases with manual curation as it might be very hard for the scientists to estimate in advance the amount of usage that they will need for a project. Moreover, this implementation is not compatible with the principles of open access. Yet, in its recent experience of the application of a subscription model, TAIR claims to minimize these drawbacks by balancing subscriber-only privileges and the publication of special releases which can be downloaded and reused by other data resources. TAIR is currently also supported by a grant from the SLOAN Foundation, with the aim of developping a suitable and advanced platform for the extension of the user funding model to other databases ^11^. Another possibility could also be to charge a modest fee to the users and to compensate the missing funds with other mechanisms that could guarantee the accessibility to the resource to the largest community at almost zero access cost.

Another possible model is a *Consortium* of many biotechnology/pharma companies that contribute to the budget. Currently, there exist some joint programmes, such as the Innovative Medicines Initiative (IMI ^17^), a partnership between the European Commission and the European Federation of Pharmaceutical Industries and Associations, that could in principle also support infrastructure and data resources. However, implementing this model for more than one database (for example for all ELIXIR Core Data Resources) may hinder the negotiations with the commercial partners. Alternatively, the idea of a separate consortium for each database is likely prohibitive.

**Figure 2.**
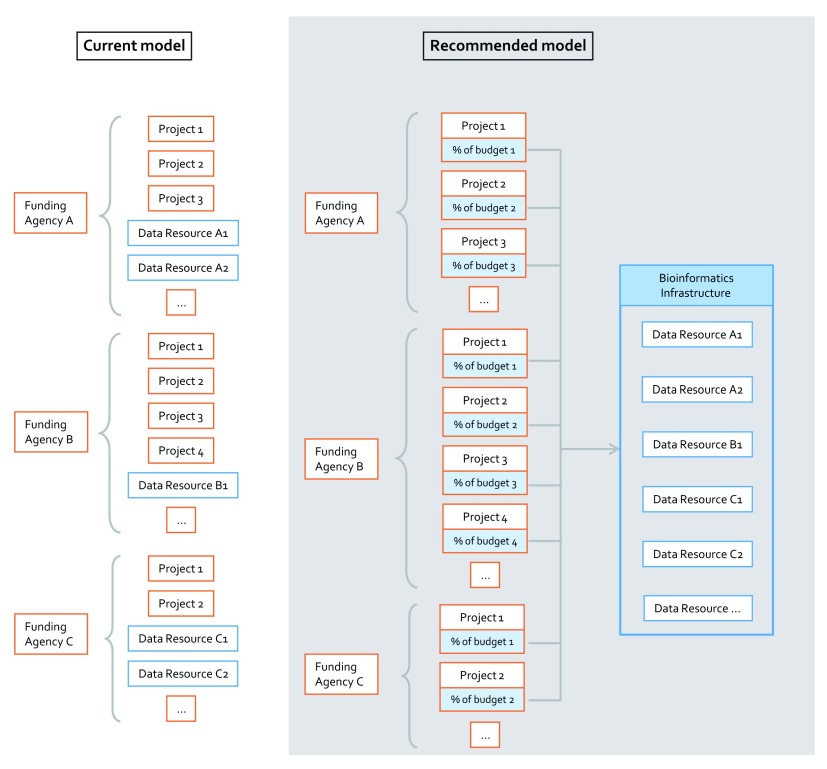
The *Infrastructure Model* on the level of the funding agency. On the left, the current model, in which databases compete cyclically for grants against research or resource projects. On the right the Infrastructure Model, in which the funding agencies distribute research grants only to research projects. A percentage of each grant is retained and assigned to a budget for data stewardship, and subsequently redistributed among the relevant infrastructures, including Data Management Plans providers, deposition databases and knowledgebases.

On the basis of these observations, in this work, the *Infrastructure Model* is proposed as a sustainable model for all the life science knowledgebases, since it is compliant with the criteria we put forward above. Its process is illustrated in Figure 2. The funding agencies distribute research grants only to research projects (but not to databases), in function of their field/topic. A percentage of each grant is retained and assigned to a budget for data stewardship and subsequently redistributed among the relevant infrastructures, including Data Management Plans providers, deposition databases and knowledgebases.

In addition, whereas UniProt’s current funding is almost entirely coming from the USA, Switzerland and the EMBL member states, this model has the advantage of distributing the cost over the countries according to the composition of the science community and thus allows a considerable diversification of the revenue streams. Importantly, at a larger scale, with less than 1 percent of the life science budget of five major funding agencies in Europe, Switzerland, Great-Britain, Japan and the USA, this model would be able to fund €190 million to cover the costs of the total core data resources. Should such a model be implemented at an even wider international level, it will involve funding agencies from other countries, thus increasing the income and further diversifying the streams. The *Infrastructure model* has also the advantage to scale with the amount of data that is generated. The implementation of such a model requires however the appointment of a *super partes* lead agency or an institution that takes care of the funding distribution process and the selection criteria. This function could be played by ELIXIR, as the European reference for the life science bioinformatics resources, or by another non-profit organization. This model could also be combined with others, such as the advertising model or some donations or institutional support.

**Figure 3.**
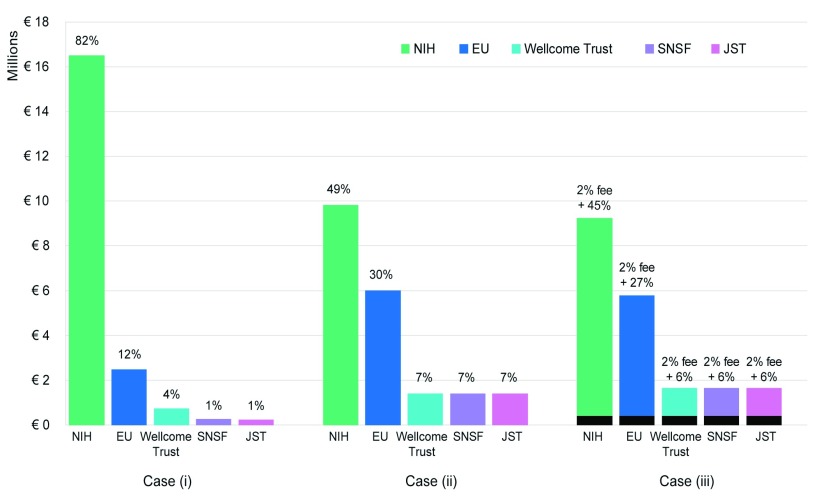
Distribution of the UniProt cost among the 5 funding agencies with the 3 variations of the *Infrastructure Model*. Case (i) is the classic model, in which the cost is covered by 0.1% of life science budget of each agency. In Case (ii), the five funding agencies are classified depending on their life science spending and total cost is shared among the categories with different percentages, but constant inside each group. In Case (iii) a fixed 2% entry fee is required from each funder (irrespective of the size) and the rest of the cost is covered by a contribution depending on the classification (S - M - L), as in Case (ii).

The *Infrastructure model* as presented could in principle be valid in the case of a consortium of funding agencies with similar volume of grants investments. However, if there is a large discrepancy among the parties, this model turns out to be unfair, as the contribution of each agency to the total budget is directly proportional to its research spending. As a consequence, “large” funders will end up in paying the largest fraction of the figure and the model will not be fair. In this situation, a variation to the model can be conceived: funding agencies are classified by size in terms of their research spending, as “small (S)”, “medium (M)” and “large (L)” funders and contribute to the total cost with a fixed percentage, depending on their category. In this way, costs will be redistributed more evenly among the funders and spread across the entire research community. A third possibility is to setup a fixed “entry fee” from each agency, that would guarantee a minimal income. The rest of the costs are distributed among the three categories of funders, again depending on their size. Figure 3 presents a comparison of the three variations of the Infrastructure Model, with the representations of the distribution of the UniProt cost among the 5 funding agencies considered for this study (NIH, EU, Wellcome Trust, SNSF, JST, see Section 4. In Case (i), each funding agency contributes with the 0.1% of its life science budget to the total cost. As the difference in research spending of the funders is so massive (the investment of the NIH into the life sciences corresponds to more than 6500% of the investment of the SNSF), the NIH ends up in paying more than 3/4 of the total cost. In Case (ii) the five funding agencies are classified depending on their life science spending and the total cost is shared among the categories: NIH as “large”, contributing for 49% of the cost, EU as “medium”, contributing for 30% of the cost and Wellcome Trust, SNSF and JST as “small”, contributing for 7% of the cost each. As clearly visible in the figure, this model has the advantage of redistributing the costs among such different funders. Case (iii) represents the extension of Case (ii), in which a fixed 2% entry fee is required from each funder (irrespective of the size) and the rest of the cost is covered by a contribution depending on the classification (S - M - L). This last variation may be perceived as the fairest, as it allows a redistribution of the costs and ensures a minimal income. The entry fee should be then set at a level that would not discourage the small funders from participating. Worldwide, similar initiatives have already been started. At the European level, the already mentioned commission High Level Expert Group on the European Open Science Cloud (HLEG-EOSC) was created in September 2015 to provide strategic advice to the European Commission on the European Open Science Cloud initiative as part of the Digital Single Market. In its discussion on the financing of research infrastructures, including e-infrastructures (e.g. ESFRI, e-IRG and Horizon 2020-related groups), the group proposed that well-budgeted data stewardship plans should be made mandatory to all research proposals and speculated that on average about 5% of research expenditure should be spent on properly managing and stewarding data. The analysis carried out in this work demonstrated that less than half of this value could actually be sufficient to maintain the core data resources.

In parallel, the USA’s NIH has launched a virtual space called Commons, a shared computing resource and a repository for data and informatics tools. In 2015, the NIH started a pilot study to test the efficacy of the Commons Cloud Credits Business Model that is designed to provide unified access to a selected choice of compute resources. In this pilot project, the researchers obtain cloud credits as part of their project grant, i.e. dollar-denominated vouchers that can be used with the cloud provider of the investigator’s choice. The cloud provider has to be Commons-compatible by meeting a set of NIH standards for capacity and capabilities. This approach is supposed to provide the researchers with a cost-effective way of accessing cloud computing resources ^22^. However, cloud providers still rely exclusively on NIH funding. Both these initiatives concern mainly digital research infrastructures, such as archives, storage and data stewardship, while discussions on curated databases are still in their infancy. The Human Frontiers Science Program Organisation (HFSPO) with the Global Life Science Data Resource Working Group, as well as the ELIXIR Long Term Sustainability Working Group and the OECD Global Science Forum project (GSF) are all working on the issue of sustainable business models for data repositories and curated databases. The HFSPO has recently proposed that an international coalition should be set up to support the core data resources in the life sciences. The coalition would first define indicators to establish the core data resources eligible for international support, develop models that provide free global access, and help assess the fraction (an estimation of 1.5/2% has been proposed) of total research funding for such resources ^27, 28^. A similar project carried out by the OECD GSF is exploring the complexity of the problems connected to the future support of the data resources in the life sciences. Discussions are based on a strong consensus that core data resources for the life sciences should be supported through coordinated international efforts that better ensure long-term sustainability and appropriately align funding allowing for access at no charge.

The model presented in this work is in line with these considerations: its approach encourages equity, internationality and economic dependability, but it necessitates major changes to the way funds are distributed. It thus requires negotiating with the funding agencies at an international level, with probably less effort than with all the user country governments, and the introduction of a suitable structure to support this model in the life science community.

## Data availability

All data required to reproduce the analysis presented in this study are included in the manuscript.

## Notes

^1^ http://www.snf.ch/SiteCollectionDocuments/Dossiers/dos_OA_regelung_auf_einen_blick_e.pdf

^2^ http://www.universitiesuk.ac.uk/policy-and-analysis/reports/Documents/2015/monitoring-the-transition-to-open-access.pdf

^3^ https://report.nih.gov/nihdatabook/charts/Default.aspx?chartId=155&catId=2, averaged on the last 10 years

^4^Communication of the Commission ‘ICT infrastructures for e-Science’ of 5.3.2009, COM(2009) 108 final

^5^ https://grants.nih.gov/grants/funding/ac_search_results.htm

^6^Corresponding to an estimated cost of the 26 candidate core data resources (5 archives, 15 knowledgebases and 6 declared as being both archive and knowledgebase, for the equivalent of 320 FTEs) submitted to ELIXIR on 1 December 2016.

^7^ https://data.oecd.org

^8^ http://p3.snf.ch/Default.aspx?id=AR2015

^9^ https://www.report.nih.gov/award/index.cfm

^10^ https://wellcome.ac.uk/funding/managing-grant/grant-funding-data-2015-2016

^11^ http://www.jst.go.jp/EN/JST_Brochure.pdf

^12^ http://ec.europa.eu/research/horizon2020/pdf/press/fact_sheet_on_horizon2020_budget.pdf,

^13^ http://www.biotechgate.com/gate/v3/statistics.php

^14^ https://data.oecd.org/entrepreneur/enterprises-by-business-size.htm#indicator-chart

^15^ https://wikimediafoundation.org/wiki/2015-2016_Fundraising_Report

^16^ https://commonfund.nih.gov/bd2k/cloudcredits

^17^ https://www.imi.europa.eu/
