# Supplementary material for: Funding knowledgebases: Towards a sustainable funding model for the UniProt use case
Source: F1000Res. 2018 Mar 22;6:ELIXIR-2051. Originally published 2017 Nov 27. [Version 2] doi: 10.12688/f1000research.12989.2 (PMC5747334; doi:10.12688/f1000research.12989.2)
Supplement: Peer review history

# Peer review: Referee response for version 1

**DOI:** 10.5256/f1000research.14085.r28422

**Published:** 2018-01-02

**Authors:** Judith A. Blake

**Affiliations:** 1 The Jackson Laboratory, Bar Harbor, ME, USA

This is a well-written and interesting approach to considering the funding of expert biological infrastructure funding. Using UniProt as the test case, 12 models of funding and sustainability are investigated and discussed. Funding of infrastructure in life sciences is an incredibly important and pressing problem as increased levels of data generation and computational analysis accessing highly structured and integrated digital data are flooding the research environment.

 The paper, far from being a generalized overview or editorial, dives deeply into an investigation of alternative funding models. It will be very useful to the user and to the funding communities to have this outline of different approaches. While I see overlap between a few of the funding mechanisms, the more important impact of the paper is that careful thought has gone into considering a range of mechanisms.  

 While adequate linkages to data resources are presented, I think it would be a useful addition to add a table of sources and the particular result from that resource that is included in the discussion. Authors state all data to reproduce are included in the study, but actually the data are extracted from external reports. That said, all data for the evaluation presented in Figure 3 are available in the study. This is a minor quibble. 

 The challenge for important, comprehensive, and extensively used resources such as UniProt, the Model Organism Databases, and others is that while they are essential infrastructure for advancement of scientific investigations, no one agency or organization wants over responsibility. The effort to globally fund digital infrastructure will require cooperation and consideration of many parties. This paper reports on the issues and possible solutions

I have read this submission. I believe that I have an appropriate level of expertise to confirm that it is of an acceptable scientific standard.

# Response

**Published:** 2018-03-18

**Authors:** Chiara Gabella

**Affiliations:** SIB Swiss Institute of Bioinformatics, Lausanne, Switzerland

We thank Dr Blake for the very positive review. We very much appreciate that the article is not perceived as a “generalized overview or editorial”, rather a call to action. We are very grateful to Dr Blake for pointing that out.

We acknowledge that some of the models might look similar (e.g. model 7 and model 8) and in fact we have joint the analysis of those two models. As it is mentioned in the text, the list is likely not exhaustive: it is rather a subjective selection of the most used funding sources for data resources. It is also worth saying again that to simplify the reading, the study is conducted as if data resources were relying on one unique funding stream. In reality, most of the resources depend on mixed models and different funding sources. Considerations should therefore be weakened and merged, by taking into account many various parameters, in order to represent the current situation. This type of analysis falls out of the scope of this work.

# Peer review: Referee response for version 1

**DOI:** 10.5256/f1000research.14085.r28421

**Published:** 2017-12-27

**Authors:** Tanya Z. Berardini, Eva Huala

**Affiliations:** 1 Phoenix Bioinformatics, Redwood City, CA, USA; 2 Arabidopsis Information Resource, Redwood City, CA, USA; 3 The Arabidopsis Information Resource, Redwood City, CA, USA

Core data resources for the global biological research community are essential for the progress of science.  Financial support for such resources is not guaranteed even given high usage and a clear need from the community.  The authors of this paper describe 12 different ways to fund a core bioinformatics data resource like UniProt and advocate the adoption of the ‘infrastructure model’ for such a purpose.

**General Comments:**

 1. The authors discuss a very important issue and raise excellent points on the value and necessity of curation and how paying for curation is not ‘paying again for already paid for information’. They also make the valid point that while many highly used resources are used globally, they are financially supported by only a fraction of their users.  An equitable distribution of the financial burden of data resource maintenance and improvement is a desirable goal.

 2. We think it would be useful to reexamine the criterion that open access equals free access.  The paper defines open-access as “digital online, free of charge, and free of most copyright and licensing restrictions”.  We propose that charging a modest fee that most of the community can afford in combination with mechanisms to enable access for those who can't afford it does not prevent access of the resource by those who need it.

**Comments on the Overview of existing funding models:**

 1) Models 7 and 8 are not clearly distinguished, these should probably be combined.

 2) Model 10,  'online advertising and corporate sponsorship', appears to us to be a mixed model.

 3) Some discussion of the 'pay to submit' model would be helpful, it is not clear why this model was not included.  Example is the Dryad Digital Repository (http://datadryad.org/).

 4) The Cambridge Crystallographic Data Centre (CCDC, https://www.ccdc.cam.ac.uk/) is an important example of the freemium model that should be included.

 5) Model 11, Open source volunteering, doesn’t belong in income generating models. Even though this is stated later in the manuscript, for clarity it should be removed from this discussion.

**Comments on the Recommendation of the Infrastructure Model**

 The proposed advantages of the infrastructure model strongly depend on the details of its implementation.  In particular, the mechanism of allocation across existing and new resources will be challenging to design in a way that is: a) fair across countries and research disciplines, b) provides sufficient support to resources across a spectrum of resources ranging from those requiring intensive curation and therefore higher cost to resources with a less intensive approach and associated lower costs, c) provides a way to reevaluate the distribution periodically and shift resources where they are most needed, d) preserves some incentive for resources to maintain high quality and serve their users well, e) in spite of the possibility for shifting resources, still enables long term planning and is stable enough to ensure long term sustainability.  In practice, this model will likely necessitate some sort of periodic evaluation of each existing or new resource to determine which will be funded and at what level; in other words, a mechanism that is very like a grant process.  Careful planning would be required to avoid the acknowledged drawbacks of the existing grant funding paradigm.  We think a discussion of some of these issues would improve the paper and enable a fairer comparison against existing funding mechanisms where the implementation details have been extensively worked out and the drawbacks are therefore more clear.

**A few points of clarification on TAIR, the resource with which we, the authors of these comments, are associated.**

 1. The paper classifies TAIR (as currently funded) as an example of the “User Subscription Fees’ model.  We think it should be considered a Mixed Model as its revenue derives in part from the National model (China and Switzerland country-level subscriptions), in part from the User Subscription Fees model (academic institutional, individual, and corporate subscribers), and in part from the Foundation model (Sloan Foundation grant).  There are also elements of the Freemium model as non-subscribers have some free page views before encountering a monthly limit.

 2. The paper states, “TAIR is actually also supported by a grant from the SLOAN Foundation, which allowed the transition to the subscription-based funding model.”  TAIR had already transitioned to subscription funding before the Sloan grant was received and funding from the Sloan Foundation was “to enhance the technology behind TAIR’s subscription funding model” and “[develop] a next-generation, flexible and customizable technology platform capable of serving other databases and research resources wishing to shift to user-based funding.”

We have read this submission. We believe that we have an appropriate level of expertise to confirm that it is of an acceptable scientific standard.

# Response

**Published:** 2018-03-18

**Authors:** Chiara Gabella

**Affiliations:** SIB Swiss Institute of Bioinformatics, Lausanne, Switzerland

We thank the reviewers for the positive feedback and the constructive comments. We have addressed the major concerns raised in the report. We have corrected in the text the TAIR description and classification and added as an example of mixed model. We are grateful for those clarifications on TAIR.

- We have maintained the definition of open access equals free of charge, but we acknowledge that very modest fees could not prevent access to the resource by those who need it. We have added a comment on open access in the discussion section.
- Models 7 & 8 look indeed similar (see comment to Dr Blake): the analysis is merged for the two as they entail similar limitations. The difference relies on the period of time during which the “free” version is available. While for model 7 there is no limit in time, model 8 sets a time frame for free usage (which implies that if a user do not pay, he does not access the resource). We preferred to retain the distinction between the two, while we agree that they could in principle be merged.
- Model 10 could indeed be seen as a type of mixed model. At a deeper analysis, most of the models are mixed models or can easily be combined to form mixed models (see comment to Dr Blake).  We preferred to maintain this selection and generate mixed models from the 12 described. Of course, other classifications according to different criteria could have been done.
- The “pay-to-submit” model was intentionally excluded as it is mainly a funding model for repositories. Knowledgebases, who do not rely on user data deposition, could not be funded through this model – unless merged with others-.
- The CCDC has been included, thank you for this precious suggestion
- Model 11 does not generate income. We preferred to retain it in the description as it has been recently considered as a possible sustaining model for some types of resources. As stated in the discussion, we agree that it cannot be a solution for curated databases.

# Peer review: Referee response for version 1

**DOI:** 10.5256/f1000research.14085.r28423

**Published:** 2017-11-30

**Authors:** Helen Berman, John Westbrook

**Affiliations:** 1 Department of Chemistry & Chemical Biology, Center for Integrative Proteomics Research, Rutgers, The State University of New Jersey , Piscataway, NJ, USA; 2 Center for Integrative Proteomics Research, Rutgers, The State University of New Jersey, Piscataway, NJ, USA

This paper analyzes of sustainability models for knowledgebases using Uniprot as an example. It makes some important points and is definitely worthy of indexing. However, there are aspects of the presentation that could be improved. Here are some suggestions.

Introduction

- A simple statement about the differences between repositories and knowledgebases is required.
- Sustainability is a problem for all data resources not just knowledgebases.
- It would be better to make generalizations in the introduction and wait until section 3 to discuss UniProt in particular.
- After the statement about the sustainability framework there should be some discussion of other similar studies. These should include the Michigan study, the HFSP study, and the commentaries by Lorsch et al. among others. Then I suggest that there be a short statement about what this paper will cover.
- The comments in last paragraph of this section belongs in the appropriate parts of section 2. In particular, the difficulty of sustaining resources from research funding sources is a key issue facing many new and existing resources.

Overview

- This section is very clear. I think that some comments from the last paragraph of the introduction could be incorporated in the appropriate model descriptions.
- All of the data resources need references.

Funding situation of Uniprot

- The history of UniProt funding exemplifies the problems in the current sustainability models.
- Table 1 is very useful and compares well with the Michigan study. Although that study focused on domain repositories in all of science, the conclusions are similar.
- I do not understand the references given in the Infrastructure model section.
- Can any estimate be made of about further gains in automating information extraction that can be anticipated from improvements in machine learning tools and techniques? In other words, is there scope for significant future reduction in manual biocuration.
- In the concluding section, it would be useful to elaborate further on the how the availability of subsidized cyber infrastructure and services *alone *would impact the long term UniProt sustainability.

We have read this submission. We believe that we have an appropriate level of expertise to confirm that it is of an acceptable scientific standard.

# Response

**Published:** 2018-03-18

**Authors:** Chiara Gabella

**Affiliations:** SIB Swiss Institute of Bioinformatics, Lausanne, Switzerland

We want to thank the authors of the report for their interesting suggestions. We have implemented the suggestions which have improved the text presentation and the flow of the paper. We are grateful to the authors for their comments. In particular, we have:

- Enhanced the difference between repositories and knowledgebases in the introduction;
- Made the introduction as general as possible, without mention to the UniProt case
- Added statement about sustainability as problem for all data resources
- Moved the manual curation description to the appropriate paragraph and added considerations about the future of manual curation, which have also been discussed with Prof. Berman in the F1000 blog https://blog.f1000.com/2018/02/07/how-best-to-fund-knowledgebases/
- Added references to all the data resources presented in the text
- Made more specific references to the previous comparable studies that are mentioned in the discussion section.
